# Supplementary material for: The miRNA-21-5p Payload in Exosomes from M2 Macrophages Drives Tumor Cell Aggression via PTEN/Akt Signaling in Renal Cell Carcinoma
Source: Int J Mol Sci. 2022 Mar 10;23(6):3005. doi: 10.3390/ijms23063005 (PMC8949275; doi:10.3390/ijms23063005)
Supplement: Supplementary file 1 [file ijms-23-03005-s001.zip › Caption of supplementary figures.pdf]

Caption of supplementary figures

Figure S1. Morphology of THP-1-Mφ and THP-1-M2 cells. Scale bar, 100 μm.

Figure S2. (**A, B**) Transwell assays of 786-O and ACHN cells treated with conditioned media derived from different macrophage subtypes. Scale bar, 100 μm. * $p < 0.05$, ** $p < 0.01$, *** $p < 0.001$, **** $p < 0.0001$. Mφ-CM: media conditioned by THP-1-Mφ cells; M2-CM: media conditioned by THP-1-M2 cells.

Figure S3. The gross view (right) of CAM tumors (left) and three representative tumors in each group are shown.
