# Supplementary material for: The miRNA-21-5p Payload in Exosomes from M2 Macrophages Drives Tumor Cell Aggression via PTEN/Akt Signaling in Renal Cell Carcinoma
Source: Int J Mol Sci. 2022 Mar 10;23(6):3005. doi: 10.3390/ijms23063005 (PMC8949275; doi:10.3390/ijms23063005)
Supplement: Supplementary file 1 [file ijms-23-03005-s001.zip › Table S1.pdf]

Table S1. Sequences of primers

Gene	Primer	Sequence
CD163	F	TTTGTCAACTTGAGTCCCTTCAC
	R	TCCCGCTACACTTGTTTTCAC
CD206	F	GGGTTGCTATCACTCTCTATGC
	R	TTTCTTGTCTGTTGCCGTAGTT
HLA-DR	F	GCCTCTTCTCAAGCACTGGGA
	R	CCACCAGACCCACAGTCAGG
TNF-α	F	CCTGTAGCCCACGTCGTAGC
	R	AGCAATGACTCCAAAGTAGACC
GAPDH	F	GGAGCGAGATCCCTCCAAAAT
	R	GGCTGITGTCATACTTCTCATGG
Vimentin	F	TCCAAGTTTGCTGACCTCTC
	R	TCAACGGCAAAGTTCTCTTC
MMP-9	F	TCGAACTTTGACAGCGACAAG
	R	TCAGGGCGAGGACCATAGAGG
PTEN	F	ACGACGGGAAGACAAGTTCA
	R	AGGTTTCCTCTGGTCCTGGT
PTEN-3‘UTR WT	F	TCGAGCTACCCCTTTGCACTTGTGGCAAC AGATAAGTTTGCAGTTGGCTAAGAGGC
	R	GGCCGCCTCTTAGCCAACTGCAAACTTAT CTGTTGCCACAAGTGCAAAGGGGTAGGC
PTEN-3‘UTR Mut	F	TCGAGCTACCCCTTTGCACTTGTGGCAAC AGTTTACTTTGCAGTTGGCTAAGAGGC
	R	GGCCGCCTCTTAGCCAACTGCAAAGTAAA CTGTTGCCACAAGTGCAAAGGGGTAGGC
miR-21-5p	Stem-loop	GTCGTATCCAGTGCAGGGTCCGAGGTATT CGCACTGGATACGACTCAACA
	F	GCACCTAGCTTATCAGACTGA
	R	GTGCAGGGTCCGAGGT
miR-155-5p	Stem-loop	CTCAACTGGTGTCGTGGGGCAATTCAGTT GAGCCCCTATC
	F	TGCCTCCAACTGACTCCTAC
	R	GCGAGCACAGAATAATACGAC
miR-210-3p	Stem-loop	GTCGTATCCAGTGCAGGGTCCGAGGTATT CGCACTGGATACGACTCAGCC
	F	TGCTGTGCGTGTGACAG
	R	GTGCAGGGTCCGAGGT
U6	Stem-loop	ACGCTTCACGAATTTGCGTGTC
	F	TCGCTTGGCAGCACATATACT
	R	ACGCTTCACGAATTTGCGTGTC

* F: Forward primer (5'-3'); R: Reverse primer (5'-3').
