# Supplementary figures and images for: The miRNA-21-5p Payload in Exosomes from M2 Macrophages Drives Tumor Cell Aggression via PTEN/Akt Signaling in Renal Cell Carcinoma
Source: Int J Mol Sci. 2022 Mar 10;23(6):3005. doi: 10.3390/ijms23063005 (PMC8949275; doi:10.3390/ijms23063005)

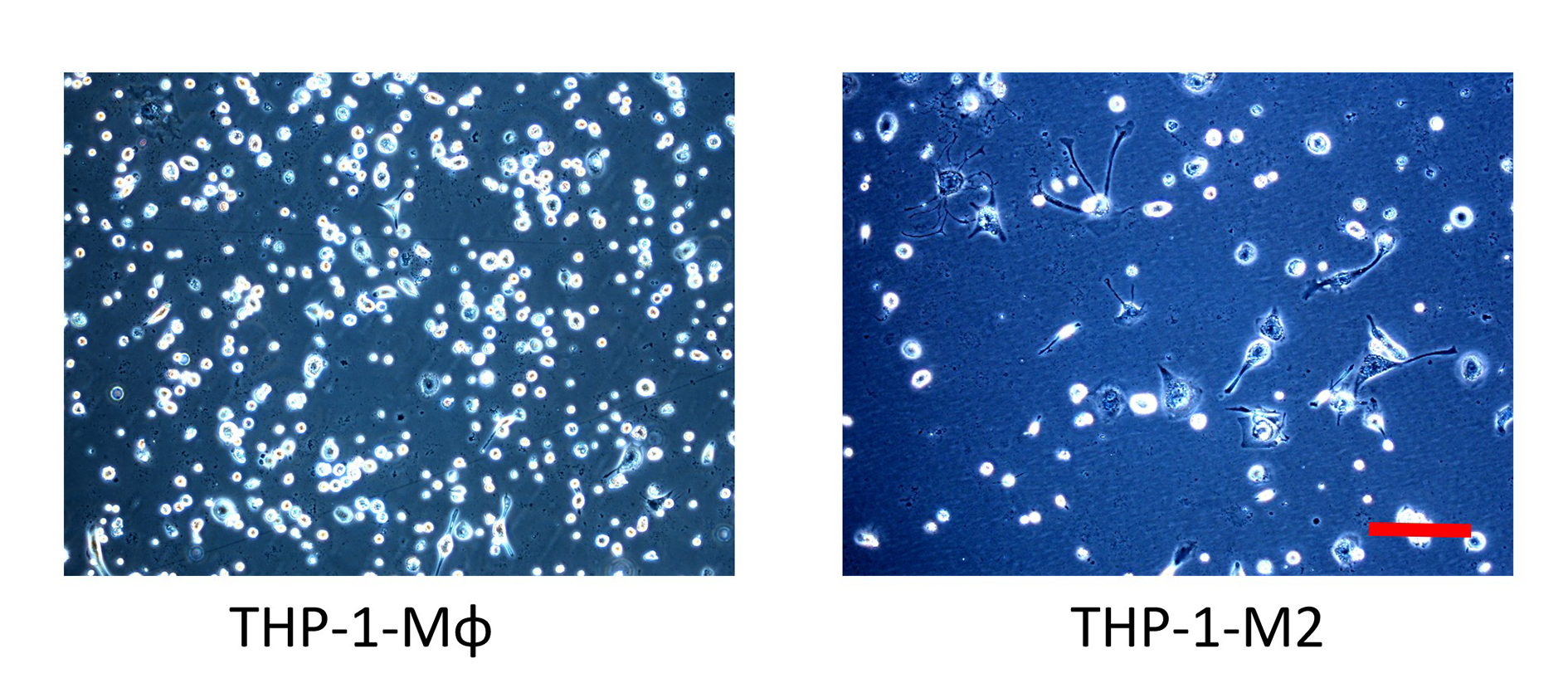

Supplement: Supplementary file 1 [file ijms-23-03005-s001.zip › Figure S1.tif]

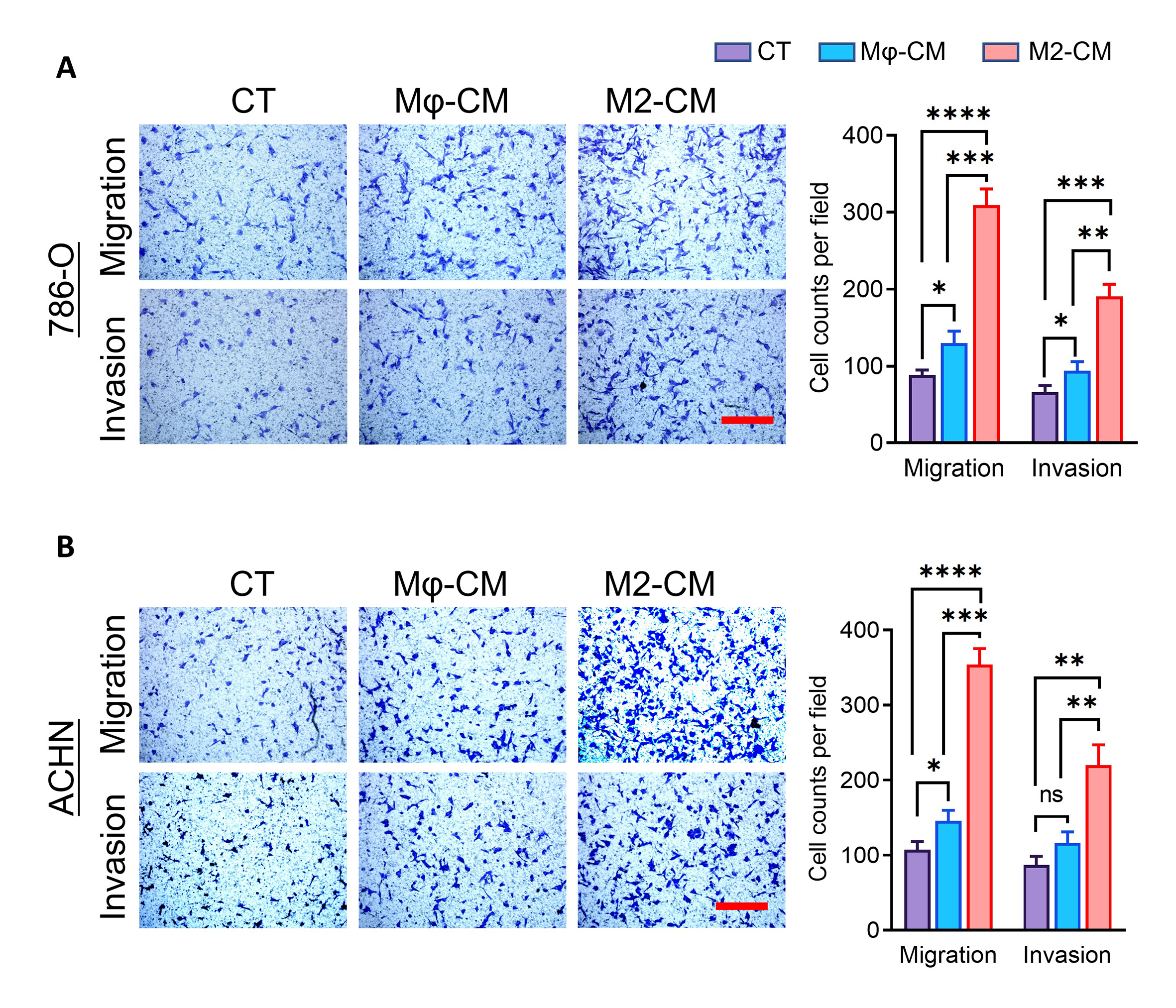

Supplement: Supplementary file 1 [file ijms-23-03005-s001.zip › Figure S2.tif]

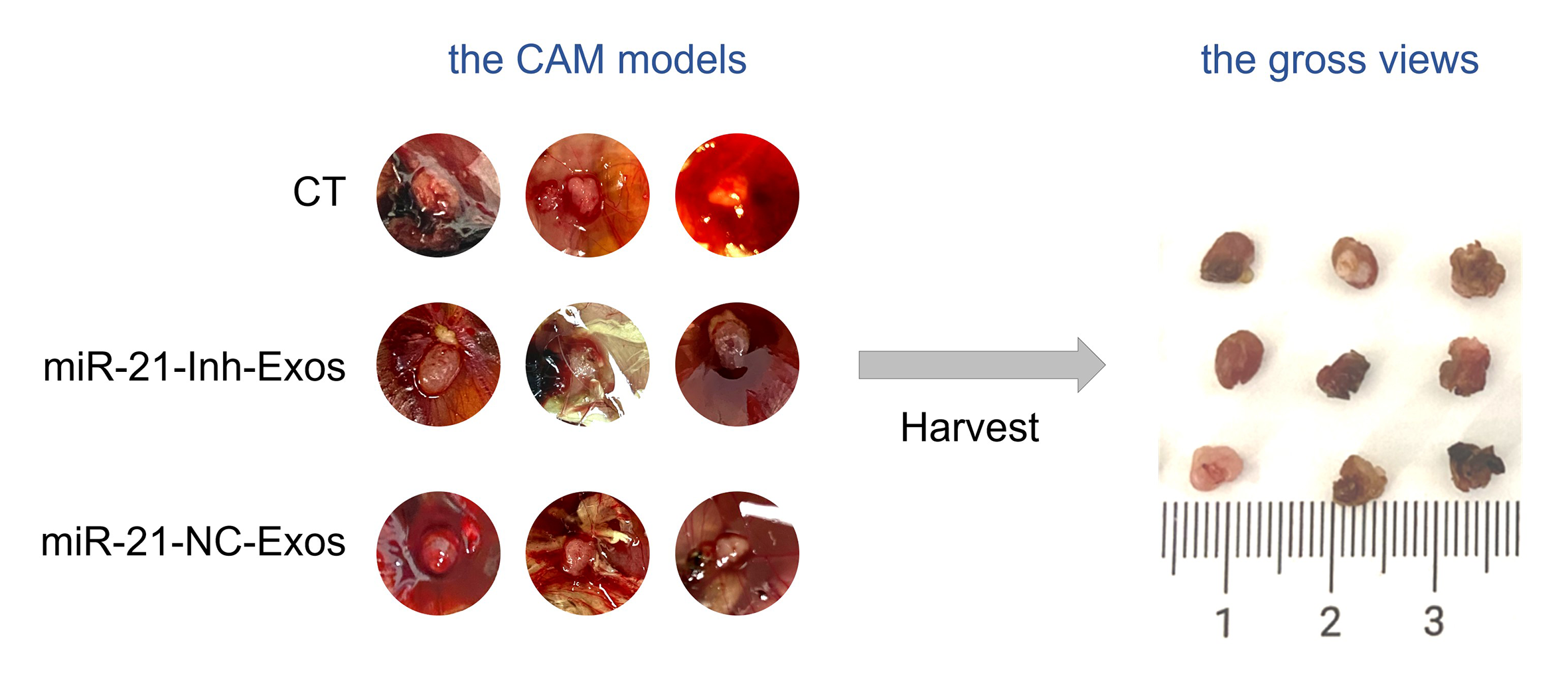

Supplement: Supplementary file 1 [file ijms-23-03005-s001.zip › Figure S3.tif]
